# Radiotherapy for gynecologic cancer in nonagenarian patients: a framework for new paradigms

**DOI:** 10.1186/s40880-016-0104-4

**Published:** 2016-05-09

**Authors:** Benoîte Méry, Sylvie Mengue Ndong, Jean-Baptiste Guy, Avi Assouline, Alexander T. Falk, Anaïs Valeille, Jane-Chloé Trone, Romain Rivoirard, Pierre Auberdiac, Alexis Vallard, Sophie Espenel, Guillaume Moriceau, Olivier Collard, Claire Bosacki, Jean-Philippe Jacquin, Guy de Laroche, Pierre Fournel, Cyrus Chargari, Nicolas Magné

**Affiliations:** Department of Medical Oncology, Lucien Neuwirth Cancer Institute, 42271 Saint Priest En Jarez, France; Department of Radiotherapy, Lucien Neuwirth Cancer Institute, 42271 Saint Priest En Jarez, France; Department of Radiotherapy, Porte De Saint Cloud Clinical Center, 92100 Boulogne-Billancourt, France; Department of Radiotherapy, Claude Bernard Private Hospital, 81000 Albi, France; Department of Radiotherapy, Val-De-Grâce Military Hospital, 75230 Paris, France; Department of Radiation Oncology, Antoine Lacassagne Center, 06100 Nice, France

**Keywords:** Gynecologic cancer, Nonagenarians, Female genital tract, Radiotherapy, Geriatrics

## Abstract

No consensus exists regarding the role of radiotherapy in the management of gynecologic cancer in nonagenarian patients. We retrospectively reviewed the outcomes of 19 consecutive nonagenarian patients with gynecologic cancer (6 endometrial cancers, 6 cervical cancers, 4 vulvar cancers, and 3 vaginal cancers) who were treated with radiotherapy. Radiotherapy was performed mainly in a palliative setting (*n* = 12; 63.2%), with a median dose of 45 Gy (range, 6–76 Gy). Infrequent major acute or late toxicities were reported. Among 19 patients, 9 (47.4%) experienced tumor progression, 5 (26.3%) experienced complete response, 2 (10.5%) experienced stable disease and/or partial response. At last follow-up, 12 patients (63.2%) had died; most deaths (*n* = 9) occurred because of the cancer. These results suggest that radiotherapy is feasible in the treatment of nonagenarian patients with gynecologic cancer.

## Background

Radiotherapy is a cornerstone in the management of gynecologic cancer. Because patients 65 years of age or older are often excluded from clinical trials, little is known about the therapeutic index (efficacy/toxicity ratio) of radiotherapy in the geriatric population [1]. For patients at high risk of local recurrence or who have unresectable or locally advanced disease, radiotherapy can be performed with the intent to cure [2–6]. Radiotherapy can also be performed in a palliative setting. A few studies reported radiotherapy-caused complications in nonagenarian patients, but data on efficacy are still scarce. The objective of the present study was to report efficacy and toxicity data on the radiotherapy treatment of nonagenarian patients with gynecologic cancer.

## Patients and methods

This retrospective study was conducted at two public and two private comprehensive cancer centers in France. Institutional review boards approved the study, which was conducted in compliance with the Declaration of Helsinki.

### Patient population

We reviewed the medical records of consecutive nonagenarian patients who received external-beam radiotherapy for the treatment of gynecologic cancer between 2003 and 2012. Patient, tumor, and radiotherapy characteristics were analyzed. The total biological equivalent dose in 2-Gy fractions (EQD2) was calculated using the linear quadratic model and an alpha/beta ratio of 10 Gy for tumors.

### Toxicity evaluation

Patients were assessed for toxicity every week during the radiotherapy treatment and every 6 months thereafter. Toxicities were graded using the Common Terminology Criteria for Adverse Events (version 4.0). Toxicities that occurred within 6 months of the beginning of radiotherapy treatment were considered acute toxicities; toxicities that occurred after 6 months were considered late toxicities. Follow-up and survival durations were calculated from receipt of the last radiotherapy fraction.

## Results

### Patient characteristics

We analyzed the data of 19 nonagenarian patients with gynecologic cancer. At the time of radiotherapy treatment, the patients’ median age was 91.4 years (range, 90.0–98.6 years). Before radiotherapy, 11 patients (57.9%) had an Eastern Cooperative Oncology Group Performance Status score of 2 or higher. Nine patients (47.4%) were nursing home residents. Among the 19 patients, histologic diagnosis revealed 6 (31.6%) endometrial cancers, 6 (31.6%) cervical cancers, 4 (21.0%) vulvar cancer, and 3 (15.8%) vaginal cancers; primarily 15 (78.9%) with a locally advanced tumor (T3–T4 or N+) or metastatic disease. Seven patients (36.8%) underwent surgery before radiotherapy, and one patient (5.3%) had previously received radiotherapy for the same indication. Patient characteristics are shown in Table 1.

**Table 1:** Characteristics of 19 nonagenarian patients with gynecologic cancer

Characteristic	No. of patients (%)
PS score	
0–1	8 (42.1)
2	5 (26.3)
3	6 (31.6)
Living place	
Home	9 (47.4)
Institution	9 (47.4)
Not reported	1 (5.2)
Primary site	
Endometrium	6 (31.6)
Cervix	6 (31.6)
Vulva	4 (21.0)
Vagina	3 (15.8)
Stage	
Localized (T1–T2 N0)	5 (26.3)
Locally advanced (T3–T4 or N+)	10 (52.7)
Metastatic	2 (10.5)
Not reported	2 (10.5)

*PS* performance status; *N*+ positive lymph node

### Radiotherapy characteristics

Three-dimensional conformal radiotherapy was used for palliation, and intensity-modulated radiotherapy was used for curative intent. The median delivered dose was 45 Gy (range, 6–76 Gy), and the median EQD2 was 44.2 Gy_α/β = 10_ (range, 8–84 Gy_α/β = 10_). The median number of fractions was 18 (range, 1–36 fractions), and the median dose per fraction was 3 Gy (range, 1.5–6 Gy). Palliative treatments were mainly hemostatic radiotherapy (8 of 19, 42.1%), followed by decompression radiotherapy (1 of 19, 5.3%) and postoperative radiotherapy (1 of 19, 5.3%); the intent of radiotherapy was not reported in two patients (10.5%). Radiotherapy was performed with the intent to cure for seven patients (36.8%).

### Efficacy and toxicity data

Median follow-up time was 18 weeks (range, 0–116 weeks). At last follow-up, seven patients (36.8%) achieved tumor control (defined as stable disease and/or partial response and/or complete response), including five complete responses; 12 patients (63.2%) treated with radiotherapy for palliative intent died, mainly because of disease progression (*n* = 9; 47.4%).

One patient who underwent 15 Gy of irradiation (total prescribed dose: 50 Gy) developed grade 5 bladder perforation and peritonitis and died after surgery for stage IV endometrial cancer); this represented 8.3% (1 of 12) of all deaths. One patient (5.3%) who underwent 44 Gy of irradiation (total prescribed dose for cervical cancer: 44 Gy) developed grade 4 diarrhea, and one patient (5.3%) developed grade 3 diarrhea. Two patients (10.5%) developed grade 2 asthenia, and one patient (5.3%) developed grade 2 epithelitis. There were two (10.5%) treatment disruptions: one caused by grade 5 toxicity, the other caused by grade 3 toxicity. One late grade 2 toxicity was observed (lower limb edema).

## Discussion

Based on these real-life findings, radiotherapy seems feasible in nonagenarian patients, either with intent to cure or in palliative setting. In this study, we retrospectively assessed the safety and efficacy of radiotherapy for 19 nonagenarian patients with gynecologic cancer. We observed only infrequent infield toxicities (5.3% grade 5, 5.3% grade 4, and 5.3% grade 3). At last follow-up, disease was controlled in 36.8% of the patients. Currently, little is known about the therapeutic index (efficacy/toxicity ratio) of radiotherapy in patients 65 years of age or older. Toxicities in small populations of geriatric patients were reported, but these studies employed outdated radiotherapy techniques [7, 8]. To our knowledge, our study is one of the largest to analyze the effects of radiotherapy on nonagenarian patients with gynecologic cancer. As expected, hypofractionated techniques were widely used, since they reduce acute toxicities (in cell populations with a high turnover, such as mucosal membranes) and favor radiotherapy completion [9]. Our results suggest that radiotherapy is feasible in nonagenarian patients with gynecologic cancer, but geriatric assessment could probably reduce treatment disruption and death rates. Although no consensus exists regarding the role of brachytherapy in the management of gynecologic cancer in patients 65 years of age or older, it should probably be considered a favorable option for geriatric patients because of its good results in terms of efficacy [10–12] and, when compared to external-beam radiotherapy, the fact that it causes fewer toxicities [11].
